# The Impact of *Pinus koraiensis* Leaf Extract Consumption on Postprandial ApoB100 and Lipid Metabolism: A Randomized, Double-Blind, Placebo-Controlled Trial in Healthy Participants Subjected to an Oral High-Fat Challenge

**DOI:** 10.3390/nu16172864

**Published:** 2024-08-27

**Authors:** Soo-yeon Park, Tae gwon Park, Kwanyong Choi, Kyeong Jin Kim, Ji Yeon Kim

**Affiliations:** 1 Department of Food Science and Biotechnology, Seoul National University of Science and Technology, Seoul 01811, Republic of Korea; sooyeon.park@seoultech.ac.kr (S.-y.P.); 21510169@seoultech.ac.kr (T.g.P.); gdragonchoi@seoultech.ac.kr (K.C.); 2 Department of Nano Bio Engineering, Seoul National University of Science and Technology, Seoul 01811, Republic of Korea

**Keywords:** *Pinus koraiensis*, Korean pine, postprandial ApoB100, lipid metabolism, oral high-fat challenge, randomized clinical trial

## Abstract

*Pinus koraiensis* (PK) leaf extract, derived from Korean pine byproducts, holds promise for alleviating postprandial hyperlipidemia. In this study, we investigated the potential of PK leaf extract for modulating postprandial hyperlipidemia in adults with normal or borderline fasting triglyceride levels. In a randomized, double-blind, parallel design, 70 subjects were randomly assigned to either the placebo or PK group for 4 weeks. After 4 weeks of consuming PK leaf extract, the results indicated a trend toward decreased serum apolipoprotein B-100 (ApoB100) levels 2 h after a high-fat challenge. Furthermore, significant improvements were observed in the incremental area under the curve (iAUC) at 0–4 h and 2–4 h compared to baseline, particularly among individuals with a higher body weight (>61.35 kg) and daily caloric intake (>1276.5 kcal). Based on these findings, PK leaf extract may have beneficial effects on postprandial lipoprotein metabolism, especially among individuals with a relatively high body weight and caloric intake.

## 1. Introduction

Cardiovascular disease (CVD) poses a significant global health challenge and is the leading cause of mortality and morbidity worldwide [1,2]. The prevalence of CVD is projected to increase in the coming years, necessitating the development of innovative strategies to combat atherosclerotic CVD [3]. Atherosclerosis, the primary pathological process contributing to CVD, involves the progressive accumulation of fibrous plaques within large- and medium-sized arteries, obstructing blood flow and increasing the risk of adverse cardiovascular events [4]. Central to atherosclerosis is the retention of cholesterol-rich lipoproteins, particularly low-density lipoprotein (LDL) and other apolipoprotein B (ApoB)-containing particles, within the arterial wall, initiating an inflammatory cascade that promotes lesion development [5].

Apolipoproteins are crucial constituents of plasma lipoproteins that exert a profound influence on vascular health and the development of atherosclerosis by modulating lipoprotein metabolism [6]. Key apolipoproteins involved in lipid metabolism and the development of atherogenesis include ApoB100, ApoB48, ApoAI, ApoCII, ApoCIII, ApoE, and Apo(a). ApoB100 serves as the primary structural component of very low-density lipoprotein (VLDL), intermediate-density lipoprotein (IDL), low-density lipoprotein (LDL), and lipoprotein(a) [7]. With projections indicating a substantial increase in cardiovascular-related mortality by 2030, urgent action is warranted to mitigate the societal and economic impacts of these diseases [8].

*Pinus koraiensis* (PK), commonly known as Korean pine, is predominantly found across northeastern Asia and its surrounding regions [9,10]. The needles and bark of *Pinus* species contain various flavonoids and phenolic acids, including quercetin, catechin, vanillic acid, pinocembrin, and pinobanksin [11,12,13]. However, following harvesting, only the pine nuts are utilized for consumption, while the other byproducts are discarded, leading to environmental pollution. This underscores the necessity for research into effective recycling methods [14]. These byproducts contain various bioactive compounds, indicating their high value when utilized as resources, as they can be recycled to reduce environmental pollution emissions.

Therefore, in this study, we hypothesized that the extract derived from PK leaves, a byproduct of Korean pine, may be beneficial for alleviating postprandial hyperlipidemia. To validate this hypothesis, this study aimed to investigate whether PK leaf extract reduces postprandial triglyceride (TG) levels in adults with normal or borderline fasting TG concentrations. Additionally, we intended to distinguish between responders and non-responders within this population to PK leaf extract and to evaluate the safety of PK leaf extract consumption. This investigation involved functional evaluation, safety assessment following consumption, and the identification of responders among participants who consumed PK leaf extract in a randomized, double-blind, parallel design trial over a 4-week period.

## 2. Materials and Methods

### 2.1. Sample Preparation

PK ethanol extract was kindly provided by Dainnatural Co., Ltd. (Cheonan, Korea). In summary, Korean pine needles were combined with 10 volumes of 50% ethanol. Following concentration, the extract was sterilized at 95 ± 5 °C for 10 min. Dextrin was then added to the sterilized concentrate at a 1:1 ratio, and the mixture was powdered. Test materials were manufactured to contain 100% Korean pine leaf extract per capsule. The placebo was formulated to emulate the taste, color, and flavor of the test material and contained crystalline cellulose, 90% caramel colorant, and 10% flavoring agent.

### 2.2. Study Design

This study was a randomized, double-blind, placebo-controlled, parallel design conducted in Bundang Jesaeng General Hospital (Gyeonggi, Republic of Korea). Participants were allocated numbers based on the order of registration at Visit 2 according to a preestablished randomization plan. Randomization was conducted using block randomization, allocating participants to the PK and placebo groups at a 1:1 ratio with an equal distribution of males and females in each group. The research protocol was approved by the Institutional Review Board of Bundang Jesaeng Hospital (DMC 2021-02-006) and registered with the World Health Organization International Clinical Trials Registry Platform (KCT 0006990).

### 2.3. Participants

Eligible participants were adults aged 20 years or older with serum TG concentrations less than 200 mg/dL. The exclusion criteria included individuals with hyperlipidemia, diabetes, hypertension, or other allergic conditions; vegetarians; individuals consuming more than 7 bottles (420 g) of alcohol per week; individuals with a body mass index (BMI) less than 22 kg/m^2^ or greater than 35 kg/m^2^; individuals who were engaging in more than 10 h of vigorous physical activity per week, pregnant, or planning to become pregnant; and individuals who had hypersensitivity to ingredients contained in the placebo or test material or who had severe food allergy reactions. Participants were recruited by posting notices on-site and advertising on subways.

### 2.4. Protocol

**Figure 1:**
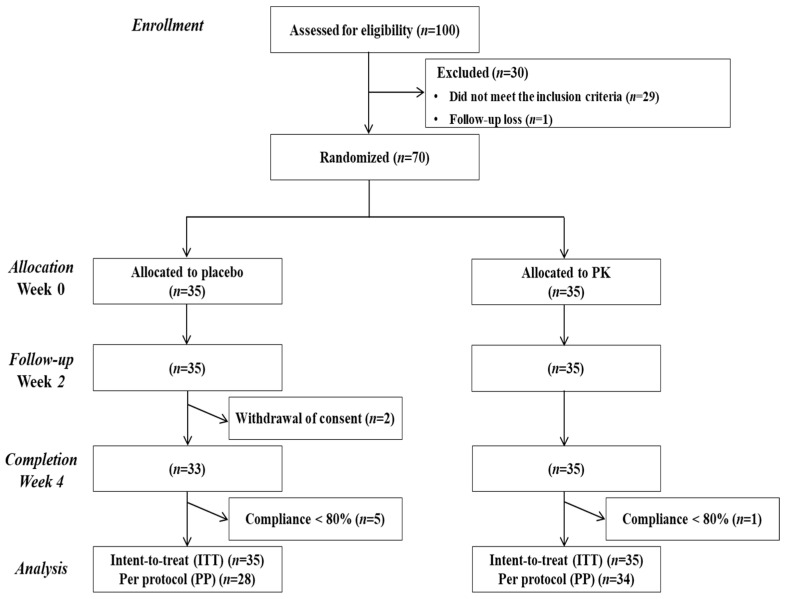
CONSORT diagram for the flow of subjects through the study.

Participants who signed the informed consent form during Visit 1 were assessed for eligibility based on the inclusion and exclusion criteria. The selected participants underwent a run-in period of 2 weeks and were then randomly assigned to either the PK group or the placebo group at Visit 2 (Week 0), according to the order of registration, and were instructed to consume 2 capsules (1000 mg/day) containing PK or placebo with water once daily for 4 weeks in a parallel design (Figure 1). Additionally, PhenFlex challenge tests were conducted by fasting for more than 12 h, followed by the consumption of a standardized TNO fat challenge formula (a formula containing 60 g of palm oil, 83.5 g of dextrose, 20 g of Protifar^®^ (protein powder, Nutricia, Utrecht, The Netherlands), and 320 mL of water) along with either the PK or placebo at Visits 2 (Week 0) and 3 (Week 4). Blood samples were collected before consumption and at 2, 4, and 6 h after consumption.

### 2.5. Assessment of Postprandial Blood Lipid Levels

After consuming the PK or placebo, blood samples were collected at 2, 4, and 6 h postprandially. Within 30 min of blood collection, the serum was separated by centrifugation at 3000× *g* for 15 min at 4 °C. The centrifuged serum was aliquoted and stored at −80 °C. TG concentrations were measured using a TG measurement reagent (Asan Pharm. Co., Anseong-si, Republic of Korea), and serum ApoB100 levels were determined according to the instructions provided with the Human ApoB100 ELISA PRO kit (Mabtech, OH, USA).

### 2.6. Statistical Analysis

An intention-to-treat analysis was conducted for clinical outcomes, and nonnormally distributed data were transformed to achieve a normal distribution before analysis. Between-group comparisons of participant characteristics at baseline were analyzed using Student’s t test for continuous variables and the chi-square test or Fisher’s exact test for categorical variables. The data are presented as the means and standard errors. The following functional variables were analyzed: For postprandial lipids, changes over time at 0 and 4 weeks were analyzed, and the incremental area under the curve (iAUC) was calculated using the trapezoidal rule. Values exceeding 1.5 times the interquartile range (IQR) from the first or third quartiles were considered outliers and excluded from the analysis. Linear mixed-effects models were used for between-group and within-group comparisons, adjusting for significant confounding factors (age). Furthermore, to clarify the effect of the PK leaf extract, machine-learning algorithms based on R software version 4.1.2 were utilized to identify responder and non-responder participants based on the positive effects of PK leaf extract consumption. Baseline characteristics of the responders, i.e., effect modifiers, were selected. The significance of functional evaluation variables was evaluated using linear mixed-effects models for responders and non-responders based on effect modifiers. A *p* value < 0.05 was considered to indicate statistical significance. Statistical analyses were performed using SAS version 9.4.

## 3. Results

### 3.1. Subject Characteristics

A total of 100 participants were screened, of whom 70 met the inclusion criteria and were allocated randomly, with 35 each in the placebo and PK treatment groups. Two participants from the placebo group withdrew consent, resulting in a final total of 68 participants (Figure 1). The baseline characteristics are presented in Table 1. The mean age was 40.8 ± 2.1 years in the placebo group and 45.9 ± 1.7 years in the PK group. Fasting serum TG levels were 75.6 ± 9.0 mg/dL in the placebo group and 78.9 ± 8.5 mg/dL in the PK group, with no significant differences observed between the groups for any of the parameters.

### 3.2. Effect of PK on Postprandial TG and ApoB100 Levels

**Figure 2:**
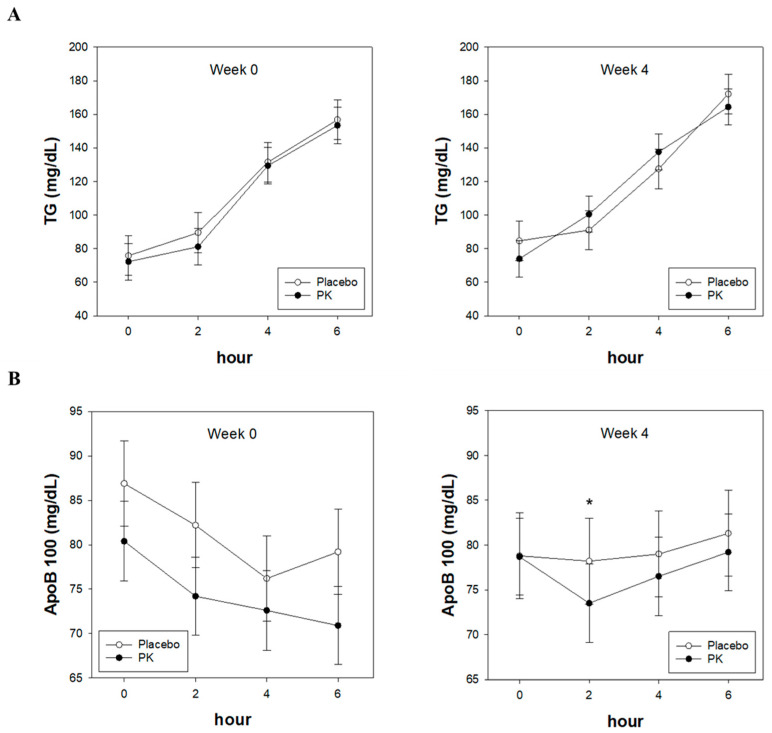

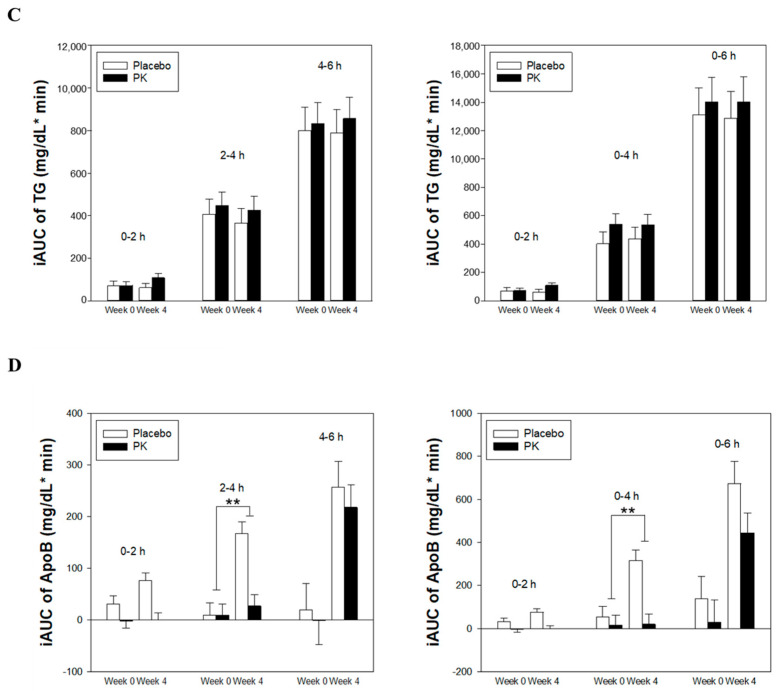
Effect of PK extract on postprandial (**A**) TG, (**B**) ApoB100, (**C**) iAUC of TG, and (**D**) iAUC of ApoB100 levels in response to high-fat challenge in Week 0 and 4. PK, *Pinus koraiensis*; TG, triglyceride; ApoB100, apolipoprotein B−100; iAUC, the incremental area under the curve. Each line represents the LS mean ± SE. *p* values were obtained from a linear mixed-effects model (adjusted for age) across the 4-week study period. * *p* value < 0.1, ** *p* value < 0.05.

The postprandial TG analysis results were consistent with those presented in Figure 2A, showing no significant differences between the groups. Figure 2A specifically illustrates the comparison of postprandial TG levels between the PK and placebo groups, with results indicating no statistically significant variations. Similarly, the postprandial ApoB100 analysis results, as shown in Figure 2B, indicated no significant differences between the groups overall.

However, in comparison to the placebo group, the PK group exhibited the following trends: There were no significant differences in serum ApoB100 levels at baseline across all time points, while at 4 weeks, a decreasing trend was observed at 2 h (*p* = 0.061), with no significant differences noted at other time points. Additionally, the iAUC analysis revealed significant reductions at 2–4 h (iAUC 2–4 h, *p* = 0.004) following a single administration of PK and high-fat challenge, as well as at 0–4 h (iAUC 0–4 h, *p* = 0.011) compared to baseline after 4 weeks of PK consumption, as depicted in Figure 2D. Figure 2D specifically illustrates these reductions in iAUC, highlighting the significant impact observed at these time intervals, which suggests a potential beneficial effect of PK consumption on postprandial lipid metabolism.

### 3.3. Qualitative Interaction Tree (QUINT) Analysis of ApoB100 Levels between Responders and Non-Responders

**Figure 3:**
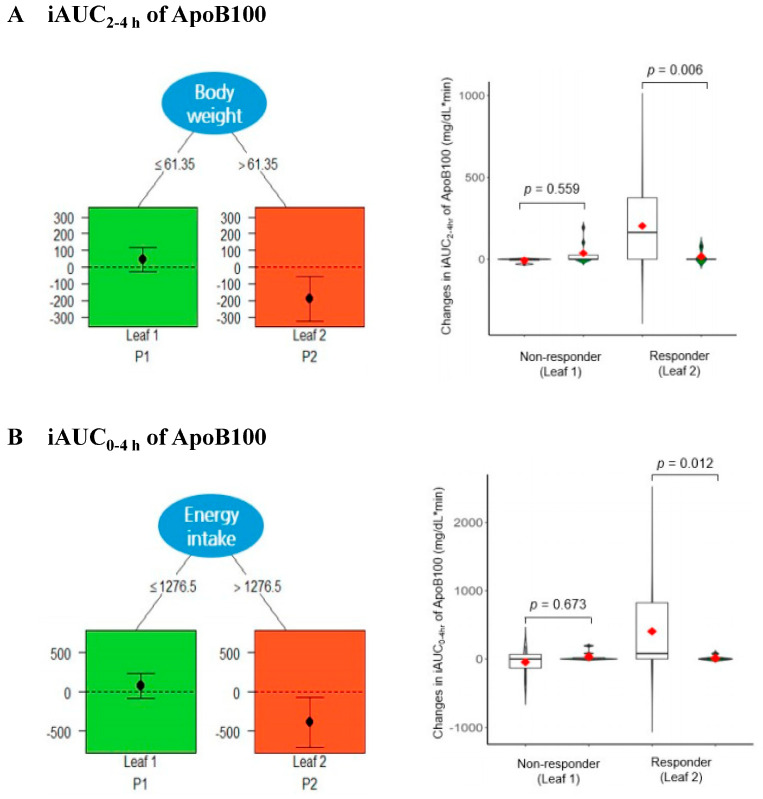
Pruned qualitative interaction tree for the outcome of QUINT analysis. (**A**) iAUC 2−4 h of ApoB100 with moderator variable “body weight” and a split point of 61.35 kg. (**B**) iAUC 0−4 h of ApoB100 with moderator variable “energy intake” and a split point of 1276.5 kcal. PK is more effective for the red leaves than placebo and the reverse is valid for the green leaves. Violin plots compare the distribution of the placebo (gray) and PK (green) groups. The interquartile range and median are shown by the vertical bar and black dash, respectively, and the mean is shown by the red dot. *p* values were obtained from a linear mixed-effects model. PK, *Pinus koraiensis*; ApoB100, apolipoprotein B−100; iAUC, the incremental area under the curve; QUINT, qualitative interaction tree.

An analysis of responders and non-responders was conducted to identify the effects of PK leaf extract consumption according to baseline weight and daily caloric intake. Among individuals with a relatively high weight (>61.35 kg), the iAUC of ApoB100 from 2 to 4 h significantly decreased in the PK group compared to the placebo group (Figure 3A, Leaf 2; responders; *p* = 0.006), whereas no significant difference was observed among those with a relatively low weight (≤61.35 kg) (Figure 3A, Leaf 1; non-responders; *p* = 0.559).

Similarly, among participants with relatively high daily caloric intake levels (≥1276.5 kcal), the iAUC of ApoB100 from 0 to 4 h significantly decreased in the PK group compared to the placebo group (Figure 3B, Leaf 2; responders; *p* = 0.012), while no significant difference was noted among those with relatively low caloric intake levels (<1276.5 kcal) (Figure 3B, Leaf 1; non-responders; *p* = 0.673).

### 3.4. Correlation Analysis of Postprandial TG and ApoB100 Concentrations

**Figure 4:**
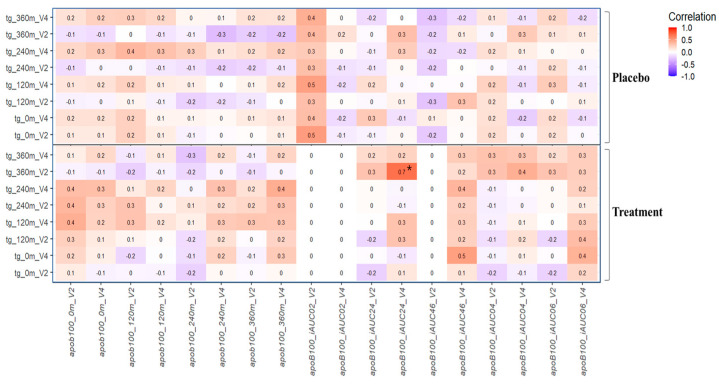
Correlation analysis of the postprandial TG and ApoB100 responses of the placebo and PK group. Pairwise Pearson correlation r values are displayed as a heatmap. Red represents a positive correlation, and blue represents a negative correlation. * *p* value < 0.05.

Correlation analysis of the postprandial TG and ApoB100 concentrations in the PK and placebo groups is shown in Figure 4. Pearson’s r values (between −1.0 and 1.0) were visualized using a heatmap. Red represents a positive correlation, and blue represents a negative correlation. The ApoB100 levels and the iAUC of ApoB100 were used for the analysis to determine the correlation between the serum concentration of TG, a strong positive correlation (r = 0.70, *p* < 0.001) was observed between the 6 h TG concentration at Visit 2 and the 2–4 h iAUC of the ApoB100 concentration at Visit 4.

## 4. Discussion

This study aimed to investigate the effects of PK leaf extract over a four-week period and lipid-loaded beverages on postprandial lipoprotein profiles and safety in individuals with normal or borderline levels of serum TG.

Neumen introduced the concept of oral lipid loading as a means to investigate the metabolism of chylomicrons [15]. Following the consumption of dietary lipids, there is a notable increase in endogenous oxidative stress levels and lipoproteins [16,17]. This increase is linked to reactive oxygen species and proinflammatory mediators produced by mononuclear cells [18]. Studies investigating the postprandial response to high-fat/high-energy beverage consumption (954.1 kcal) in healthy individuals have shown elevated blood pressure, heart rate, and intracellular adhesion molecule-1 (ICAM-1) and interleukin (IL)-8 concentrations [19]. Such dietary habits, characterized by an excessive fat intake, contribute to the development of atherosclerosis, a life-threatening arterial condition. They induce endothelial dysfunction, platelet activation, and other significant alterations in vascular physiology [20,21]. An increase in postprandial TG is directly associated with reduced VLDL cholesterol, while other lipoproteins reportedly possess the ability to activate platelets [22,23]. With chronic excessive fat intake known to promote atherosclerosis and metabolic disorders associated with coronary artery disease, the importance of regulating postprandial TG is underscored [24]. Postprandially, increases in both TG and ApoB100 concentrations were observed following consumption of the high-fat challenge in this study. Single postprandial TG levels at 4 and 6 h after the high-fat challenge significantly increased, along with the extent of the area under the TG curve at 6 h post-challenge, as shown in Figure 2.

ApoB is the predominant protein found in TG-rich lipoproteins and exists in two forms: ApoB48 in the intestine and ApoB100 in the liver. VLDL, which is synthesized in the liver and contains ApoB100, is converted into its remnants, IDL and LDL. ApoB100 has been implicated in the formation of atherogenic plaques [25]. An increase in the ApoB100 concentration signifies an increase in LDL and plays a crucial role in the occurrence of obesity and CVDs [26]. According to previous studies, compared with LDL, ApoB100 demonstrates a greater sensitivity and specificity in predicting CVDs regardless of age and sex [27]. In our study, the postprandial ApoB100 concentration showed a decreasing trend at 4 weeks and 2 h (*p* = 0.061), with no significant differences observed at other time points. There was a significant reduction in the iAUC at 2–4 h following a single administration of PK and high-fat challenge, as well as at 0–4 h, compared to baseline after 4 weeks of PK consumption. This result indicates that postprandial ApoB levels were altered following the consumption of PK leaf extract.

Pine species contain approximately 15% pinolenic acid, a polyunsaturated fatty acid (PUFA) which is recognized for its role in reducing triacylglycerol levels through various mechanisms, including inhibiting lipid synthesis, decreasing substrate availability for lipoprotein formation, and altering the physical and chemical characteristics of VLDL. As a result, the consumption of PK may lead to decreased triacylglycerol and VLDL concentrations, potentially reducing the risk of CVD [28]. In a human intervention study involving individuals with borderline dyslipidemia, daily consumption of pine leaf extract, including the bark, at a dose of 1200 mg for 12 weeks resulted in a significant decrease in the VLDL cholesterol concentration and a significant increase in SOD activity compared to those in the placebo group after consumption [29].

Previous studies have highlighted that the leaves of *Pinus* species are rich in polyphenols such as quercetin, catechin, ferulic acid, and caffeic acid, which are bioactive substances known for their antioxidant properties and potential health benefits [30,31]. Additionally, pinene, a monoterpene commonly found in *Pinus* species, is recognized not only for its historical use in flavor and fragrance production but also for its broad spectrum of biological activities, including fungicidal, insecticidal, anti-inflammatory, antioxidative, and neuroprotective effects [32,33]. Recent research by Lee et al. has shown that lemon essential oil (*Citrus limon* (L.)), which includes α-pinene, induces a hypocholesterolemic effect in rabbits. Similarly, α-pinene was observed to decrease cholesterol levels, cholesterol fractions, and TG concentrations in rats [34]. Consistent with these findings, Santos et al. reported significant hypoglycemic, hypolipidemic, and anti-inflammatory effects of α-pinene in diabetic rats, further underscoring its potential therapeutic benefits [35].

The decreased ApoB100 concentration observed in this study was particularly pronounced in the analysis identifying responders (individuals for whom an intervention is beneficial) and non-responders (those for whom an intervention has no benefit) [36]. Specifically, significant reductions in postprandial ApoB100 levels were noted after the consumption of PK leaf extract, particularly among subjects with a body weight exceeding 61.35 kg and an energy intake exceeding 1276.5 kcal/day. For participants with a body weight of 61.35 kg or greater, the average BMI was 25.9 kg/m^2^, indicating that the subjects were classified as overweight [37]. These results are comparable to studies in which a significant positive correlation was observed between cholesterol, TG, or total beta lipoprotein levels and relative body weight [38]. Considering that body weight and caloric intake serve as indicators influenced by various factors and accounting for their ease of assessment and lack of bias, correlations with lipid markers can provide supplementary insights from various studies. This study demonstrates that PK leaf extract not only influences ApoB100 but also affects other lipoprotein markers, highlighting its potential impact on lipid metabolism. However, the study was limited in its ability to thoroughly explore the underlying mechanisms and signaling pathways associated with these effects. To address this limitation and provide a deeper understanding of how PK leaf extract modulates lipid metabolism, further studies are necessary. Future research should specifically investigate the signaling pathways involved in the observed effects of PK leaf extract on ApoB100. One potential approach could involve treating cells with varying concentrations of PK leaf extract and analyzing changes in key signaling pathway proteins, including their phosphorylation status and expression levels. Additionally, to further elucidate the effects of PK leaf extract on other lipoprotein markers, lipidomics analysis using LC-MS/MS could be conducted. This approach would allow for a comprehensive assessment of changes in various lipoprotein markers, including LDL, HDL, and VLDL, providing a more detailed understanding of the physiological impacts of PK leaf extract on lipid metabolism. By addressing these research questions, future studies can contribute to a more complete understanding of the potential mechanisms and therapeutic implications of PK leaf extract in lipid metabolism and related diseases. These insights could pave the way for the development of more targeted and effective treatments using PK leaf extract or its active components.

## 5. Conclusions

In conclusion, the results of this study suggest that PK leaf extract may have beneficial effects on postprandial lipoprotein metabolism, especially among individuals with a relatively high body weight and caloric intake. Importantly, no significant adverse reactions or notable changes were observed in the safety parameters throughout the study, indicating the safety of PK leaf extract consumption (Table S1).

## Figures and Tables

**Table 1:** Baseline characteristics ^1^.

Variables	Placebo			PK			*p* Value ^2^
Age (yr)	40.8	±	2.1	45.9	±	1.7	0.064
Sex (male/female)	13	/	15	13	/	21	0.515
Menstrual status (Y/N/NA)	12/	3	/13	13/	8	/13	0.417
Alcohol consumption status (Y/N)	12	/	16	19	/	15	0.307
Alcohol consumption volume (SD/wk)	4.4	±	1.4	5.6	±	1.5	0.545
Smoking status (Y/N)	6	/	22	3	/	31	0.277
Smoking amount (cigarette/d)	1.9	±	0.7	0.6	±	0.4	0.142
Body weight (kg)	70.4	±	2.3	68.4	±	1.9	0.488
BMI (kg/m^2^)	25.2	±	0.4	25.2	±	0.4	0.991
Waist circumference (cm)	87.1	±	1.9	86.2	±	1.4	0.722
Blood lipid profile (mg/dL)							
TG	75.6	±	9.0	78.9	±	8.5	0.794
TC	208.5	±	7.4	211.2	±	6.7	0.789
ApoB	89.0	±	3.4	91.2	±	3.4	0.662
ApoB100	86.3	±	5.2	84.3	±	5.5	0.798

^1^ Mean ± SE (all such values). PK, *Pinus koraiensis*; NA, not applicable; SD, standard drink; BMI, body mass index; TG, triglyceride; TC, total cholesterol; ApoB, apolipoprotein B. ^2^ Student’s *t* test for continuous variables and the chi-square test or Fisher’s exact test for categorical variables were used to compare the differences between the groups.

## Data Availability

The original contributions presented in the study are included in the article/Supplementary Material, further inquiries can be directed to the corresponding author.

## Supplementary Materials

The following supporting information can be downloaded at https://www.mdpi.com/article/10.3390/nu16172864/s1, Table S1: Vital sign, Table S2: Hematological test; Table S3: Blood chemistry test; Table S4: Urine test; Table S5: Normal range for hematological test, blood chemistry test and urine test; Table S6: Adverse event.
