# Supplementary material for: The Impact of Pinus koraiensis Leaf Extract Consumption on Postprandial ApoB100 and Lipid Metabolism: A Randomized, Double-Blind, Placebo-Controlled Trial in Healthy Participants Subjected to an Oral High-Fat Challenge
Source: Nutrients. 2024 Aug 27;16(17):2864. doi: 10.3390/nu16172864 (PMC11397107; doi:10.3390/nu16172864)
Supplement: Supplementary file 1 [file nutrients-16-02864-s001.zip › nutrients-3147558-supplementary.pdf]

Supplementary Table

Table S1. Vital sign [1]

Variables	Placebo	PK	*p* value [2]		
			Group	Time	Group*time
SBP (mmHg)					
Week 0	125.1 ± 2.3	125.9 ± 2.3			
Week 4	126.9 ± 2.4	123.6 ± 2.3	0.682	0.847	0.121
p value [3]	0.340	0.211			
DBP (mmHg)					
Week 0	78.9 ± 1.8	76.4 ± 1.8			
Week 4	79.3 ± 1.8	77.8 ± 1.8	0.377	0.402	0.628
p value [3]	0.804	0.345			
Pulse rate (beats/min)					
Week 0	80.5 ± 1.7	75.1 ± 1.7			
Week 4	82.7 ± 1.8	77.6 ± 1.7	0.025	0.019	0.847
p value [3]	0.128	0.067			
Body temperature (°C)					
Week 0	35.9 ± 0.1	36.0 ± 0.1			
Week 4	36.1 ± 0.1	36.1 ± 0.1	0.623	0.030	0.905
p value [3]	0.105	0.139			

[1] LSmean ± SE (all such values). PK, *Pinus koraiensis*; SBP, systolic blood pressure; DBP, diastolic blood pressure.
[2] Linear mixed-effect model was used to analyze the effects of group, week and group*week.
3 Linear mixed-effect model was used to analyze the difference within each group.

Table S2. Hematological test [1]

Variables	Placebo	PK	*p* value [2]		
			Group	Time	Group*time
WBC (10^3/mm^3)					
Week 0	6.1 ± 0.2	5.3 ± 0.2			
Week 4	5.6 ± 0.2	5.2 ± 0.2	0.028	0.029	0.222
p value [3]	0.018	0.475			
RBC (10^6/mm^3)					
Week 0	4.6 ± 0.1	4.7 ± 0.1			
Week 4	4.6 ± 0.1	4.6 ± 0.1	0.969	0.586	0.703
p value [3]	0.909	0.507			
Hb (g/dL)					
Week 0	14.0 ± 0.2	14.1 ± 0.2			
Week 4	14.0 ± 0.2	13.9 ± 0.2	0.852	0.140	0.265
p value [3]	0.797	0.065			
Hct (%)					
Week 0	42.4 ± 0.6	42.3 ± 0.6			
Week 4	42.4 ± 0.6	42.2 ± 0.6	0.827	0.922	0.752
p value [3]	0.880	0.767			
MCV (fL)					
Week 0	91.4 ± 0.7	90.9 ± 0.7			
Week 4	91.6 ± 0.7	91.2 ± 0.7	0.643	0.185	0.997
p value [3]	0.355	0.338			
MCH (pg)					
Week 0	30.3 ± 0.3	30.3 ± 0.3			
Week 4	30.2 ± 0.3	30.0 ± 0.3	0.757	0.069	0.189
p value [3]	0.717	0.026			
MCHC (g/dL)					
Week 0	33.1 ± 0.1	33.3 ± 0.1			
Week 4	33.0 ± 0.1	32.9 ± 0.1	0.890	0.009	0.233
p value [3]	0.297	0.007			
PLT (10^3/mm^3)					
Week 0	259.9 ± 9.1	245.1 ± 9.1			
Week 4	264.7 ± 9.1	253.9 ± 9.1	0.309	0.027	0.508
p value [3]	0.270	0.040			

Table S2. Hematological test [1] (**continued**)

**Variables**	**Placebo**	**PK**	***p* value [2]**		
			**Group**	**Time**	**Group*time**
Neutrophils (%)					
Week 0	56.0 ± 1.2	52.5 ± 1.2			
Week 4	56.3 ± 1.2	53.7 ± 1.2	0.049	0.304	0.542
p value [3]	0.769	0.242			
Lymphocytes (%)					
Week 0	34.6 ± 1.1	37.7 ± 1.1			
Week 4	34.1 ± 1.1	36.2 ± 1.1	0.062	0.165	0.500
p value [3]	0.614	0.141			
Monocytes (%)					
Week 0	6.5 ± 0.3	6.5 ± 0.3			
Week 4	6.5 ± 0.3	6.6 ± 0.3	0.854	0.920	0.574
p value [3]	0.747	0.635			
Eosinophils (%)					
Week 0	2.3 ± 0.3	2.8 ± 0.3			
Week 4	2.6 ± 0.3	2.9 ± 0.3	0.346	0.076	0.374
p value [3]	0.064	0.517			
Basophils (%)					
Week 0	0.6 ± 0.0	0.6 ± 0.0			
Week 4	0.6 ± 0.1	0.6 ± 0.0	0.774	0.753	0.647
p value [3]	0.920	0.581			

[1] LSmean ± SE (all such values). PK, *Pinus koraiensis*; WBC, white blood cell; RBC, red blood cell; Hb, hemoglobin; Hct, hematocrit; MCV, mean corpuscular volume; MCH, mean corpuscular hemoglobin; MCHC, mean corpuscular hemoglobin concentration; PLT, platelet.

[2] Linear mixed-effect model was used to analyze the effects of group, week and group*week.

[3] Linear mixed-effect model was used to analyze the difference within each group.

Table S3. Blood chemistry test [1]

Variables	Placebo	PK	*p* value [2]		
			Group	Time	Group*time
ALT (IU/L)					
Week 0	20.1 ± 2.2	20.7 ± 2.2			
Week 4	19.1 ± 2.2	22.6 ± 2.2	0.474	0.698	0.256
p value [3]	0.598	0.275			
AST (IU/L)					
Week 0	20.4 ± 1.4	21.3 ± 1.4			
Week 4	19.5 ± 1.4	22.4 ± 1.4	0.251	0.939	0.272
p value [3]	0.473	0.399			
GGT (IU/L)					
Week 0	26.3 ± 5.0	34.1 ± 5.0			
Week 4	26.1 ± 5.0	33.8 ± 5.0	0.268	0.794	0.986
p value [3]	0.865	0.842			
Alkaline phosphatase (IU/L)					
Week 0	64.9 ± 3.9	62.9 ± 3.9			
Week 4	64.5 ± 4.0	64.9 ± 3.9	0.876	0.593	0.414
p value [3]	0.842	0.333			
BUN (mg/dL)					
Week 0	14.1 ± 0.6	14.7 ± 0.6			
Week 4	13.3 ± 0.6	13.5 ± 0.6	0.650	0.012	0.646
p value [3]	0.145	0.033			
Creatinine (mg/dL)					
Week 0	0.8 ± 0.0	0.8 ± 0.0			
Week 4	0.8 ± 0.0	0.8 ± 0.0	0.456	0.476	0.678
p value [3]	0.432	0.830			
Total bilirubin (mg/dL)					
Week 0	0.7 ± 0.1	0.7 ± 0.1			
Week 4	0.7 ± 0.1	0.7 ± 0.1	0.937	0.851	0.416
p value [3]	0.662	0.473			
Uric acid (mg/dL)					
Week 0	5.5 ± 0.2	5.1 ± 0.2			
Week 4	5.2 ± 0.2	5.0 ± 0.2	0.316	0.036	0.337
p value [3]	0.034	0.405			
Total protein (g/dL)					
Week 0	7.3 ± 0.1	7.2 ± 0.1			
Week 4	7.3 ± 0.1	7.2 ± 0.1	0.253	0.883	0.911
p value [3]	0.980	0.853			
Albumin (g/dL)					
Week 0	4.6 ± 0.0	4.6 ± 0.0			
Week 4	4.7 ± 0.0	4.6 ± 0.0	0.419	0.423	0.907
p value [3]	0.631	0.511			

Table S3. Blood chemistry test [1] (**continued**)

**Variables**	**Placebo**	**PK**	***p* value [2]**		
			**Group**	**Time**	**Group*time**
Glucose (mg/dL)					
Week 0	97.2 ± 2.2	101.1 ± 2.2			
Week 4	99.7 ± 2.2	102.3 ± 2.2	0.251	0.166	0.598
p value [3]	0.182	0.535			
eGFR (mL/min/1.73m^2)					
Week 0	96.2 ± 2.5	97.3 ± 2.5			
Week 4	97.2 ± 2.6	97.1 ± 2.5	0.879	0.718	0.628
p value [3]	0.555	0.930			

[1] LSmean ± SE (all such values). PK, *Pinus koraiensis*; ALT, alanine aminotransferase; AST, aspartate aminotransferase; GGT, gamma-glutamyl transferase; BUN, blood urea nitrogen; eGFR, estimated glomerular filtration rate.

[2] Linear mixed-effect model was used to analyze the effects of group, week and group*week.

[3] Linear mixed-effect model was used to analyze the difference within each group.

Table S4. Urine test [1]

Variables	Placebo		PK		*p* value [2]
	NCS	CS	NCS	CS	
pH					
Week 0	35	0	35	0	-
Week 4	33	0	35	0	-
p value [3]	-		-		
Protein					
Week 0	35	0	35	0	-
Week 4	33	0	35	0	-
p value [3]	-		-		
Glucose					
Week 0	35	0	35	0	-
Week 4	33	0	35	0	-
p value [3]	-		-		
Ketone					
Week 0	35	0	35	0	-
Week 4	33	0	35	0	-
p value [3]	-		-		
Blood					
Week 0	35	0	35	0	-
Week 4	33	0	35	0	-
p value [3]	-		-		
Urobilinogen					
Week 0	35	0	35	0	-
Week 4	33	0	35	0	-
p value [3]	-		-		
Bilirubin					
Week 0	35	0	35	0	-
Week 4	33	0	35	0	-
p value [3]	-		-		
Nitrite					
Week 0	35	0	35	0	-
Week 4	33	0	35	0	-
p value [3]	-		-		
Leukocyte					
Week 0	35	0	35	0	-
Week 4	33	0	35	0	-
p value [3]	-		-		
Specific gravity					
Week 0	35	0	35	0	-
Week 4	33	0	35	0	-
p value [3]	-		-		

[1] Number of subjects. PK, *Pinus koraiensis*; NCS, not clinically significant; CS, clinically significant.

[2] Fisher's exact test was used to compare the difference between the groups and p-values were not computed because CS column contains all zeros.

[3] McNemar's test was used to compare the difference within each group and p-values were not computed because CS column contains all zeros.

Table S5. Normal range for hematological test, blood chemistry test and urine test [1]

Variables	Normal range
Hematological test	
WBC ($10^3/mm^3$)	4.0-10.0
RBC ($10^6/mm^3$)	M: 4.10-6.30 / F: 3.80-5.40
Hb (g/dL)	M: 13.0-17.0 / F: 12.0-16.0
Hct (%)	M: 36.0-55.0 / F: 33.0-51.0
MCV (fL)	M: 81.0-101.0 / F: 79.0-100.0
MCH (pg)	M: 27.0-33.0 / F: 26.0-32.0
MCHC (g/dL)	32.0-36.0
PLT ($10^3/mm^3$)	150-400
Neutrophils (%)	40.0-80.0
Lymphocytes (%)	20.0-50.0
Monocytes (%)	2.0-12.0
Eosinophils (%)	0.0-8.0
Basophils (%)	0.0-2.0
Blood chemistry test	
ALT (IU/L)	0-40
AST (IU/L)	0-40
GGT (IU/L)	M: 15-101 / F: 9-42
Alkaline phosphatase (IU/L)	M: 40-129 / F: 35-104
BUN (mg/dL)	8.0-22.0
Creatinine (mg/dL)	0.80-1.50
Total bilirubin (mg/dL)	M: 0.1-1.5 / F: 0.2-1.4
Uric acid (mg/dL)	3.0-8.0
Total protein (g/dL)	6.0-8.0
Albumin (g/dL)	3.5-5.2
Glucose (mg/dL)	60-110
eGFR (mL/min/1.73m^2)	60.0-1000.0
Urine test	
pH	4.5-8.0
Protein	Negative
Glucose	Negative
Ketone	Negative
Blood	Negative
Urobilinogen (E.U./dL)	0.0-1.0
Bilirubin	Negative
Nitrate	Negative
Leukocyte	Negative
Specific gravity	1.005-1.030

[1] Normal ranges are from Bundang Jesaeng General Hospital. WBC, white blood cell; RBC, red blood cell; Hb, hemoglobin; Hct, hematocrit; MCV, mean corpuscular volume; MCH, mean corpuscular hemoglobin; MCHC, mean corpuscular hemoglobin concentration; PLT, platelet; ALT, alanine aminotransferase; AST, aspartate aminotransferase; GGT, gamma-glutamyl transferase; BUN, blood urea nitrogen; eGFR, estimated glomerular filtration rate.

Table S6. Adverse event [1]

Variables	Placebo	PK	*p* value [2]
**Total AEs**			
AE	2 / 3	2 / 4	1.000
SAE	0 / 0	0 / 0	-
**Events**			
Enteritis	1 / 1	1 / 1	1.000
Diarrhea	0 / 0	1 / 1	1.000
Dyspepsia	1 / 1	0 / 0	1.000
Stomach cramps	1 / 1	0 / 0	1.000
Nausea	0 / 0	1 / 1	1.000
Rotator cuff tear	0 / 0	1 / 1	1.000
**Severity**			
Mild	2 / 3	2 / 3	1.000
Moderate	0 / 0	1 / 1	1.000
Severe	0 / 0	0 / 0	-
**Relation to treatment**			
Definitively related	0 / 0	0 / 0	-
Probably related	0 / 0	0 / 0	-
Possibly related	0 / 0	1 / 1	1.000
Unlikely to be related	2 / 2	2 / 3	1.000
Unrelated	1 / 1	0 / 0	1.000
Unknown	0 / 0	0 / 0	-

[1] Number of subjects / number of cases. *PK, Pinus koraiensis*; AE, adverse event; SAE, serious adverse event.

[2] Fisher's exact test was used to compare the difference of numbers of subjects between the groups.
